# Obstruction of the Bowel

**Published:** 1907-06

**Authors:** Marshall Clinton

**Affiliations:** Buffalo, N. Y.; 516 Franklin Street


### BUFFALO MEDICAL JOURNAL.

VOL. LXII. JUNE, 1907. No. 11

### ORIGINAL COMMUNICATIONS.

### Obstruction of the Bowel.

By MARSHALL CLINTON, M.D., Buffalo, N. Y.

IN meeting with cases of obstruction of the bowel the physician is always confronted with the question as to whether the condition is one depending upon some acute inflammatory process, or due to some mechanical obstruction, gradual or sudden in its onset. Stenosis of the intestine or gradual narrowing of the lumen of the intestine is of slow development. Among the common causes of stenosis of the intestine we find stricture as a result of cicatricial contraction, malignant disease or, more rarely, benign growths within the bowel and compression from without, as pressure by a tumor.

Irrespective of the development of the stenosis itself we find that the symptoms may develop very gradually or may occur suddenly when the patient is in apparent health. The primary cause of all the fundamental symptoms of narrowing of the lumen is interference with the onward passage of intestinal contents. As soon as the passage of bowel contents becomes difficult, so that the normal peristaltic wave is insufficient to force these contents through the point of constriction, symptoms appear. The severity of the symptoms and the time of their appearance will depend largely on the location of the stenosis, as we know that almost complete obstruction may exist in the small intestine before any symptoms are noticed, while slight narrowing of the lumen of the large intestine, particularly the latter half, will be followed by early symptoms.

In stenosis of the large intestine and rectum constipation is an early symptom; gradually this becomes more intractable and patients complain of a feeling of distention and swelling in the abdomen; then of loss of appetite and occasional attacks of nausea. One of the characteristic early symptoms of stenosis of the large intestine are these alternating attacks of constipation followed by diarrhea. After a time, irrespective of the location of the stenosis, paroxysmal attacks of colic occur. The time in the development of stenosis that colic appears varies widely, as hypertrophy of the bowel wall above the stenosed area will delay the onset of this symptom. Many patients complain of colic as the first symptom. The colic may be localised or diffused over the abdomen and even the chest, the colic being frequently accompanied by vomiting.

In all cases during paroxysms of colic absolute constipation exists. If the abdomen of a patient with stenosis be examined during a paroxysm, tonic contraction of the intestine, above the stenosis, may be seen and felt. The picture present on inspection of the abdomen is typical of this condition—that is, coils of intestine stiff from tetanic contraction, which rise above the level of the abdomen. The attacks of colic may recur after a few days or not for many weeks. When stenosis is far advanced and the attacks recur with great frequency, the abdomen remains permanently distended and the patient suffers from dyspnea. As stenosis progresses, attacks of colic occur every day until ultimately the symptom picture of complete obstruction develops. In cases of stenosis there is frequently a feeling of fluctuation on palpation, and occasionally distinct succussion sounds can be produced, particularly in the intestine above the stenosis.

When loops become much distended and filled with fluid the percussion note in the abdominal flank may be dull, changing its level with change in position of the patient as seen frequently in ascites. However, this may be differentiated from ascites by making rapid taps against the dull area, which will reveal distinct succussion sounds. To attempt to differentiate clinically between the various forms of stenosis of the bowel, is too hard a problem to be readily mastered. We know, however, that irrespective of the cause of the stenosis this condition generally tends to result in complete occlusion. Given then any case in which we find the prominent symptoms as outlined, we are reasonably sure that unless that patient is relieved, there will ultimately result an occlusion or, as it is more commonly known, an acute intestinal obstruction.

Surgical experience and medical practice show no class of cases more to be dreaded than that of acute intestinal obstruction. The mortality under any method of treatment is alarmingly high. Surgical intervention in a developed case of intestinal obstruction is the best hope these patients have; and unless this be offered to them at an earlier period than happens commonly, our statistics will not improve. No accident as a post-operative complication, except peritonitis, is so feared as this.

In the majority of post-operative obstruction cases of course a localised or general peritonitis is the initial cause, and the subsequent obstruction is but a part of the symptom complex. This is shown in every case of peritonitis that goes progressively from bad to worse. It is undoubtedly extremely hard at times to differentiate between cases of post-operative intestinal obstruction due to peritonitis, and cases in which peritonitis develops secondarily to a mechanical obstruction.

In following post-mortem work it has often chanced that a patient has been thus examined, who died of a post-operative obstructive lesion with a secondary peritonitis, when the history of that particular case showed that the patient had been treated as one suffering from a spreading peritonitis and not as a case of intestinal obstruction. It is important to remember that in every case of intestinal obstruction the symptoms are not due simply to the interruption of the flow of intestinal contents through the bowel. Complications, such as circulatory disturbances in the intestinal wall and mesentery and alterations in functions of these structures, are in large measure responsible for the symptoms seen.

As soon as the occlusion of the bowel occurs the appetite is lost, and the patient begins to complain of nausea and belch gas. Nausea persists until vomiting finally occurs. At first the stomach contents are vomited mixed with bile, and then fecal vomiting occurs. Fecal vomiting is only absent in very acute and very rapidly fatal cases of strangulation of the bowel. In these cases death ensues before the formation of feculent bodies can occur in the upper intestinal tract. The act of retching and vomiting determines whether fecal vomiting occurs and not the antiperistaltic movement of the bowel; for cases may go on to fatal termination with the entire gastrointestinal tract filled with fecal material, while no fecal vomiting may have occurred.

As an illustration of excessive retrostalsis in a case of incomplete obstruction, with a spreading peritonitis, the writer has seen an olive oil enema vomited up several hours after its injection into the rectum. With the onset of early pain which is so often seen, an evacuation of the bowels may occur, associated with a great deal of tenesmus; all of the expelled material coming from the lower bowel, beyond the point of obstruction. This symptom is often misleading as naturally an obstruction of the bowel is not suspected when a bowel movement occurs. After this movement, however, nothing further passes the bowel as the result of either cathartics or enemata, unless it be a little fecal material mixed with the enema that has over-distended the rectum. No flatus or real feces from now on are passed; the belly begins to distend, the distention depending upon the anatomic character of obstruction and its situation in the intestine.

At this time the most serious symptom may develop,—that of shock and collapse. The face of the patient becomes distorted with pain, and a frightened expression appears; extremities become cold and bluish; pulse becomes rapid, small and compressible. Gradually the gastrointestinal symptoms increase in severity and hiccough becomes violent; nausea becomes very distressing and fecal vomiting continues. In addition the patient suffers from an unquenchable thirst; the abdomen becomes more and more distended; respiration becomes more rapid and shallow, and the patient is tortured with a terrible feeling of oppression. The urinary secretion is partially or completely arrested; the patient becomes weaker and weaker, and the picture is rendered more distressing by the fact that the mental condition is almost unaffected, while experiencing great pain.

In the last stages the skin is sallow and flaccid, cool, livid, and frequently covered with a cold sweat. The eyes of the patient are sunken and surrounded by dark circles; the face looks pinched; the nose sharp and pointed; the voice weak, and the pulse feeble and threadlike.

In the early stage the degree of pain the patient suffers depends largely upon the extent of the involved intestine; the larger the amount of intestine involved in the process the greater the pain. In acute obstruction of the small intestine, as a rule, there is more pain than in obstruction of the larger gut. The pain and obstruction is constant, as in peritonitis; is increased by pressure, remits, and becomes exacerbated, due to violent peristaltic movement above the point of obstruction. When pain ceases without relief of the obstruction it indicates a fatal ending.

One symptom that varies greatly in different parts is the increase in intraabdominal pressure. This occurs as a result of the distention of the gut, and as a result of the reflex action of the abdominal and diaphragmatic muscles. This increased pressure causes an embarrassed heart action, and also what is an extremely important matter, it may prevent the intestines from regaining their normal position, by reason of peristaltic pull. It favors the development of new areas of stasis so that a compromised bowel will be held as if in a vise, by this increased pressure. Some patients cannot recover after an obstruction is relieved surgically; this being the case particularly when there is a tremendous increase of abdominal pressure, unless this, in turn, be relieved by an enterostomy or colostomy.

In differentiating between the stenosis that occurs in normal or healthy gut and that which occurs as the result of a gradual narrowing of the intestinal lumen, it should be remembered that in chronic stenosis we see the wave-like movement of energetic, resistant intestine against the belly wall; while in acute obstruction the abdominal wall will be tense, resistant, and rarely shows any intestinal movement. In intestinal strangulation the intestinal and peritoneal branches of the pneumogastric and the sympathetic are first stimulated. Irritation of these fibers leads to alteration of the heart's action and, by causing reflex paralysis of the cardiac branches of the pneumogastric and the splanchnic nerves, further produces alterations in the general distribution of the blood throughout the general circulation. When reflex paralysis of the splanchnics occurs there will be an enormous distention of the abdominal organs with blood. The temperature of the skin and the rectum is lowered until peritonitis ensues. This latter condition occurs by reason of the passage of bacteria through the wall of the intestine as soon as the bowel wall becomes edematous, or there is any interference with the local circulation.

The prognosis of acute intestinal obstruction depends on a variety of factors aside from the time it may be operated upon for relief. As a general rule, we may say that the life of these patients is not threatened so much by the interference with the onward passage of the feces and the intestinal occlusion, as by the symptom of collapse. Here is the key to the surgical prognosis of any operated case. If there is marked collapse and great increase in abdominal rigidity the patient's chances are extremely small, even if the condition has existed but a short time. On the other hand, patients may have an intestinal obstruction of several days and yet recover with operation. The most interesting although the most rapidly fatal case of acute obstruction that has come under the writer's notice occurred last August.

A young man, 26 years old, was seized with cramps in the afternoon about 2 o'clock. His condition did not improve under anodynes or enemas so about midnight he walked from his bed into an automobile, and was driven 22 miles to the hospital. He was seen by me at 4:30 A.M., after his arrival at the hospital and I found him in a state of collapse—no radial pulse; nausea, and vomiting; no abdominal rigidity, although there was slight distention and some pain on pressure. His condition did not warrant anything being done except hot packs, with small repeated doses of morphine and stimulants. His collapse did not let up for an instant and he died before noon. Total duration of illness, less than 24 hours. An immediate post-mortem showed an intussception with over two feet of strangulated ileum in the cecum. There was no peritonitis whatever, except in the peritoneum lining the invaginated intestine. This represents one of those cases of early death from collapse. . The other two causes of death are usually peritonitis or exhaustion.

As an illustration of exhaustion without peritonitis or collapse as a cause of death I might mention a case of complete stenosis of the sigmoid, occurring as a sequel to a carcinoma of the gut where the patient, for a period of six weeks, did not pass a bubble of flatus or a particle of fecal material by his rectum, and finally died from exhaustion with an empty intestinal tract that had been cleaned out by retrostalsis, assisted by daily abdominal massage.

In the report of the following cases only those that are post-operative are included. No case of intestinal obstruction that had not been previously operated for some abdominal lesion is included. No case is reported where an obstruction was suspected and a general peritonitis found. The cases are as follows:

Case 1. Aug.—1904. Italian, 26 years old, male. Had had several attacks of appendicitis, and was convalescing from such an attack on admittance to the Erie County hospital. At the operation a dense mass of old and recent adhesions were met with and a large, thick appendix removed. Portion of the ileum at the ileocecal valve and the wall of the cecum were denuded of peritoneum. He did well after the operation and was over his vomiting from his anesthetic in 8 hours. In the evening of the following day he complained of cramp like pains, and began to vomit. There was some intestinal distention and slight tenderness of the abdomen, but a normal temperature. The house surgeon called up and said he had a case of post-operative obstruction. The patient was seen, the house surgeon complimented on making a brilliant diagnosis, and the patient operated about midnight. In delivering the cecum into the wound, the ileum was found flexed on itself, with recent delicate adhesions holding the angulation so firmly that no intestinal contents could be milked past the point of obstruction. Gently freeing the bowel permitted the contents to bubble past the affected point. He was closed up tight and made a rapid and uninterrupted recovery. This is the earliest case, being less than 48 hours from the time of the original operation.

Case 2. July—1904. Boy, 12 years old. Acute perforative appendicitis. Appendectomy and drainage on account of well marked peritonitis and abscess. Fourteen days after operation, the patient developed symptoms of acute obstruction, and an immediate operation was performed. A mass of adhesions was found matting the ileum together, and after these were freed the bowel returned and the patient was closed up without drainage. No gas or feces passed after this operation and as the parents refused to permit further surgical interference, he was taken home where he died 5 days later. The operation had afforded him no relief.

Case 3. Italian, 38 years old, male, operated Dec. 28, 1904, by one of my colleagues for an acute appendicitis. The patient did not do very well after the operation and four days later he evidently had some peritonitis and a well marked obstruction. The writer was asked to operate on him and did so on the fourth day, finding a volvulus of a coil of small intestine in the right iliac fossa, bound down by recent adhesions and a well marked peritonitis. The obstruction was relieved and the case drained. He died four days later from peritonitis.

Case 4. L. E. Operated by a colleague early in Nov. 1905. Patient had considerable post-operative peritonitis, following an operation for recurrent appendicitis. The patient was left in my charge and developed pain and tenderness in the left iliac fossa, obstructive symptoms, and considerable collapse three weeks after the first operation. A diagnosis of obstruction was made, the belly opened in the left smilunaris and matted coils of ileum found through which nothing could be milked. Loosening up adhesions and freeing the coils was followed by reestablishment of the fecal current, and the abdomen was sewed up, with drainage. Recovery rapid and uneventful. In this case the collapse was the most marked feature.

Case 5. March—1904. Fred H. Admitted to the hospital with marked signs of general peritonitis, and marked collapse. Patient had been sick four days from appendicitis. Belly found full of pus and numerous abscesses with thin walls of masses of lymph. Patient drained on both sides of the abdomen, in both flanks, in the middle line, and sent to bed with the expectation that he would promptly die. Irrigation of the abdominal cavity with five gallons of hot salt solution was ordered every six hours, and patient placed in the elevated position. Forty-eight hours after this operation he had a radial pulse; and made a slow convalescence. Owing to the wide-spread peritonitis that had developed it was carefully explained to him that he might also develop an obstruction, and how it would affect him. He left the hospital and six months later arranged to return to work. That morning at 4 A.M., his brother called up on the telephone saying: "Freddy says he has got it, and wants you to send the ambulance for him right away." When he was seen at the hospital he located an obstruction at a point corresponding to McBurney's point on the left side. He was opened and I found a thick adhesion band lying across the small gut and obstructing it. Cutting this with scissors was followed by a bubble of intestinal contents, past the obstructed point, and after sewing him up he was sent to bed where he made an easy recovery.

Case 6. Feb—1906. This probably should not be included in this series as her obstruction had nothing to do with her operation. A woman, Mrs. K., 43 years old, with chronic cholelithiasis was operated by removing one large gallstone and draining the gall-bladder. She had an enormous umbilical hernia as large as her own head, which she stipulated should not be touched during the operation on her gall-bladder. Just 14 days after her gallbladder operation she developed an obstruction in the hernal sac that could be easily seen with only the skin and sac covering the intestine. She was taken back to the operating room and a radical herniotomy done after Mayo's method. Her recovery was rapid and satisfactory and she is able for the first time in twenty years to wear a straight front corset.

Case 7. July—1906. V. N. child eight years old. Acute appendicitis. Sick five days. Abscess drained. Convalescence normal for an abscess case until the 11th day, when she developed obstructive symptoms referable to the left side. A small abscess was opened and drained on the left side, and a mass of adhesions forming an obstruction in the small gut found, her condition for 24 hours was very grave, but she gradually improved and soon was enjoying her holiday vacation.

Case 8. Aug. 8, 1906. Male inmate at the Erie County penitentiary. Patient had been operated (appendectomy) in Detroit eight months previous. Two months later operated at another hospital in Detroit for relief of obscure abdominal symptoms. Said the doctors told him his bowels had grown together and they couldn't do anything for him. Has had increasing difficulty, and cramps in trying to get his bowels to act. Has been completely obstructed for 48 hours. Large scar of an old appendix operation wound and a scar from the symphysis pubes to the umbilicus. Belly distended and flaccid. A generous incision along the border of the right rectus muscle revealed a dry tubercular peritonitis, with the intestines one large mass of adhesions and tubercles. After carefully separating the distended intestines from each other, a denser mass of tubercular tissue and coils of small intestine was found marking the point of obstruction. These were carefully separated but the intestine was torn into. The coil was followed up until the gut was entirely separated, and resection of about seven inches of ileum was made that was badly involved and torn. The appendix was found in the pelvis, riddled with tubercles and removed. The incision was sewn up without drainage; and the patient not only made a good recovery from the operation but on his discharge had regained his health.

Case 9. Jan., 1906. Miss S., aged 32. A late appendix case with a localised abscess and perforated appendix; drained and she made a slow recovery, with a fecal fistula. Five weeks after the operation developed symptoms of obstruction, with localised pain and tenderness in the upper right quadrant of the abdomen, with slight fever. This obstruction seemed to be associated with some inflammatory condition as a localised peritonitis and not purely mechanical. The upper end of the appendix scar was extended upward and a coil of small intestine delivered into the wound lying around an abscess, the size of a hen's egg. The gut at this point was empty but distended above. Cleaning out the abscess cavity showed the mesentery not seriously involved so the gut was straightened out, the coil replaced, and a small drain left where it had lain. Her recovery was rapid.

In the analysis of these cases that have occurred in the writer's practice in the past three years we find two deaths. Cases two and three died, and the remaining seven recovered. This does not include any cases of intestinal obstruction, except those of a post-operative type. In other cases, such as obstruction from hernia, peritonitis with appendicitis, cancer of the intestine, etc., my records do not show any such favorable results. Only two of these operated cases show marked or even serious collapse at the time of intervention, to which the writer attributes largely the high percentage of recoveries. When collapse is marked, when there is a well marked secondary peritonitis, and tremendous increase in intraabdominal pressure, these cases are generally hopeless. It is only by a prompt recognition of existing conditions and their rapid surgical relief that a greater percentage of recoveries can be established.

516 Franklin Street.

